# Using Google Trends to Predict COVID-19 Vaccinations and Monitor Search Behaviours about Vaccines: A Retrospective Analysis of Italian Data

**DOI:** 10.3390/vaccines10010119

**Published:** 2022-01-14

**Authors:** Andrea Maugeri, Martina Barchitta, Antonella Agodi

**Affiliations:** Department of Medical and Surgical Sciences and Advanced Technologies “GF Ingrassia”, University of Catania, 95123 Catania, Italy; andrea.maugeri@unict.it (A.M.); martina.barchitta@unict.it (M.B.)

**Keywords:** COVID-19, SARS-CoV-2, vaccine, vaccinations, hesitancy

## Abstract

Google Trends data are an efficient source for analysing internet search behaviour and providing valuable insights into community dynamics and health-related problems. In this article, we aimed to evaluate if Google Trends data could help monitor the COVID-19 vaccination trend over time and if the introduction of COVID-19 vaccines modified the interest of pregnant women in vaccination. Data related to Google internet searches and the number of vaccine doses administered in Italy were used. We found moderate to strong correlations between search volumes of vaccine-related terms and the number of vaccines administered. In particular, a model based on Google Trends with a 3-week lag showed the best performance in fitting the number of COVID-19 vaccinations over time. We also observed that the introduction of COVID-19 vaccines affected the search interest for the argument “vaccination in pregnancy” both quantitatively and qualitatively. There was a significant increase in the search interest after the launch of the COVID-19 vaccination campaign in Italy. Qualitative analysis suggested that this increase was probably due to concerns about COVID-19 vaccines. Thus, our study suggests the benefits of using Google Trends data to predict the number of COVID-19 vaccine doses administered, and to monitor feelings about vaccination.

## 1. Introduction

COVID-19 vaccines were introduced between late 2020 and the first months of 2021, by launching large-scale vaccination programmes across many countries. Many of these programmes were organised in waves, prioritising people at higher risk of severe disease, such as healthcare workers, older individuals and those suffering from pre-existing conditions [1]. Since then, national and international authorities have experienced intense demand for data to monitor and manage vaccination programmes [2]. In general, vaccine uptake and delivery can be monitored through two complementary sources: administrative reporting systems and periodic surveys [2]. The first can provide information about vaccine use, adverse events following immunisation and other service-level data. However, administrative reporting systems are limited in the way they produce disaggregated data and rely on accurate reporting [2]. Periodic surveys, on the other hand, are often directed at representative samples of households and/or facilities to evaluate aspects such as immunisation outcomes and service delivery [3]. The latter, despite offering the possibility of producing more disaggregated data than administrative reporting systems, are less frequent and more expensive [3].

In this scenario, there is a need for developing innovative tools that work alongside existing sources of data. Web-based big data analytics has been gaining popularity for its potential to monitor diseases [4,5,6,7]. In particular, this field of research is widely known as infodemiology, and it concerns the use of internet-based healthcare and disease information sources [6]. Big data produced by Google Trends are an efficient source for analysing internet search behaviour, providing valuable insights into community dynamics and health-related problems. Analysis of Google Trends has proved applicable to correlation assessment and forecasting modelling in the area of infectious diseases [8,9,10,11]. 

In the last two years, Google Trends analytics has already been applied to predict COVID-19 outbreaks, forecast the epidemic and understand vaccine hesitancy and anti-vaccination attitudes [12,13,14,15]. In particular, vaccine hesitancy is proving to be an important barrier to tackling the COVID-19 pandemic through effective vaccination campaigns [16,17]. Thus, an analysis of Google search behaviours could provide valuable insights into the feelings and fears of people before receiving the vaccination. A better understanding of this issue is important, especially for certain at-risk categories. Among them, pregnant women represent one of the most vulnerable categories during the COVID-19 pandemic. 

In this article, we explored two hypotheses concerning the relationship between Google Trends data and COVID-19 vaccination. The first was that Google Trends data could help monitor vaccination trends over time by reflecting or even anticipating the number of vaccine doses administered. The second hypothesis was that Google Trends could help understand whether the introduction of COVID-19 vaccines has raised and/or modified the interest of pregnant women in the topic of vaccination. Thus, we first analysed the Google search activity of Italian people on COVID-19 vaccination to investigate the relationship between search volume and the number of COVID-19 vaccine doses administered over time. Our aim was not only to examine whether a correlation exists between Google Trends data and vaccine doses administered, but also to employ such data for modelling purposes. Next, we analysed Google search activity on vaccination during pregnancy before and after the introduction of COVID-19 vaccines. In this case, the aim was to evaluate changes in Google search behaviours associated with the launch of the COVID-19 vaccination campaign.

## 2. Materials and Methods

### 2.1. Google Trends Data

This study was conducted in accordance with the REporting of studies Conducted using Observational Routinely-collected health Data (RECORD) statement [18] and checklist (Supplementary Table S1). To examine the relationship between Google Trends and the number of vaccines administered, data related to Google internet searches in Italy were downloaded from the Google Trends website (https://trends.google.com/trends (accessed on 4 November 2021), using the following keywords: “Vaccino” (Argument), “Vaccino COVID-19” (Vaccine) and “Vaccino Covid” (Search term). From now on, we will use the respective terms translated into English: Vaccine (Argument), COVID-19 Vaccine (Vaccine) and COVID Vaccine (Search term). Keywords were selected based on their popularity on Google Trends during the study period. Data were filtered by location (Italy) for a 44-week period from 27 December 2020 to 31 October 2021. We chose this time span to cover the period from the first vaccine administration until now. 

To evaluate changes in Google search behaviours about vaccination during pregnancy, data were downloaded in the same way using the keyword “Vaccino in gravidanza” (Search term). From now on, we will use the respective term translated into English: Vaccine in pregnancy (Search term). In this case, data were filtered by location (Italy) for a 148-week period from 1 January 2019 to 31 October 2021. We chose this time span to monitor changes from two years before the introduction of COVID-19 vaccines until now. 

It is worth noting that Google Trends supplies the search interest as the relative search volume for each keyword. Thus, search interest is obtained by dividing the number of searches for a given keyword by the total searches within a specific location and period. Its value is reported as a standardised measure ranging from 0 (no search activity) to 100 (peak of search activity). For the most popular keyword, search interest at the regional level and the most common related searches were also obtained.

### 2.2. Data on the Number of COVID-19 Vaccine Doses Administered

The corresponding data on the daily number of COVID-19 vaccine doses administered during the same period were obtained from the GitHub database (https://github.com/italia/covid19-opendata-vaccini (accessed on 15 December 2021). Data were converted into weekly number of doses administered per 100,000 inhabitants and normalised in a range from 0 (i.e., no doses administered) to 100 (i.e., the maximum doses administered) to facilitate the comparison with Google Trends data.

### 2.3. Statistical Analysis

To assess the relationship between Google Trends data and the weekly number of COVID-19 vaccines administered, a cross-correlation analysis was initially performed using lag and lead Spearman correlation coefficients (Spearman’s ρ). Correlation coefficients were calculated for the most popular keyword with a maximum lag/lead time of 12 weeks. Next, autoregressive integrated moving average (ARIMA) models were applied for time series modelling of the number of COVID-19 vaccines administered in Italy. In general, an ARIMA model consists of three components (i.e., auto regression, differencing and moving average). Its general notation is ARIMA (p, d, q), where p is the autoregressive order, d is the differencing order and q is the moving average order [19]. In an ARIMA model, the dependent variable must be stationary, and this assumption can be checked by visualising the sequence chart [19]. In the present study, the time series of the weekly number of vaccine doses administered was not stationary. Thus, stationarity was achieved by calculating the first-order differencing of the time series [19]. Once the dependent variable was stationary, the parameters for p and q were determined by inspecting the autocorrelation function (ACF) and the partial autocorrelation function (PACF) [19]. Based on the ACF and PACF, different ARIMA models were evaluated, and those with a non-significant *p*-value (*p* < 0.05) were discarded. To assess the ability of Google Trends to improve the prediction of COVID-19 vaccinations, several ARIMA models with an exogenous variable (ARIMAX) were applied. The ARIMA model with the best performance was chosen as the baseline for the ARIMAX modelling [19]. Thus, ARIMAX models included the search interest of the most popular keyword as an exogenous variable, with a maximum lag time of 7 weeks. The exogenous variable underwent the same transformation process as the dependent variable. For both ARIMA and ARIMAX models, the goodness of fit was evaluated through the root mean square error (RMSE), mean absolute error (MAE) and mean average percentage (MAPE), while the residuals were inspected for the existence of white noise using the Ljung–Box test [19].

To evaluate changes in Google search behaviours concerning vaccination in pregnancy, an ARIMA model was applied to predict search volume for the selected keyword from 1 January 2021 to 31 October 2021, using observed data from 1 January 2019 to 31 December 2020. Predicted values were compared with those observed, and their differences reflected changes after the introduction of COVID-19 vaccines. 

All the statistical analyses were performed using the SPSS software (Version 26; IBM Corp., Armonk, NY, USA).

## 3. Results

### 3.1. Google Search Interest for Vaccine

**Figure 1:**
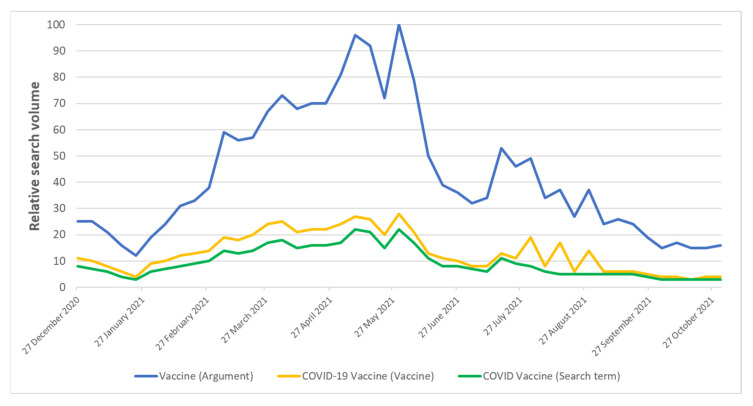
Comparison of the search interest for keywords Vaccine (Argument), COVID-19 Vaccine (Vaccine) and COVID Vaccine (Search term).

**Figure 2:**
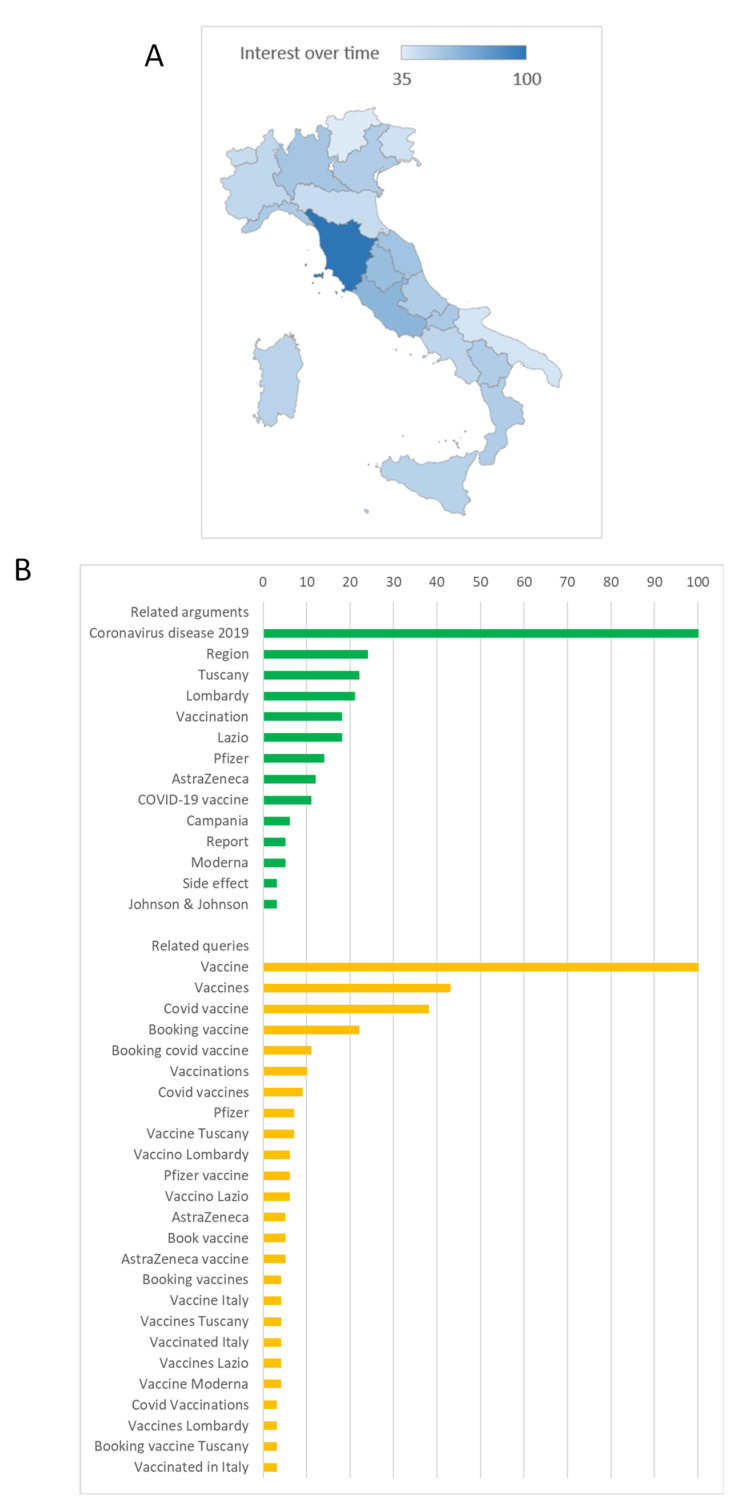
Search interest for the keyword Vaccine (Argument). (**A**) Comparison of Google Trends data across Italian regions; (**B**) the most common arguments and queries related to Vaccine (Argument).

Figure 1 reports comparisons between overall trends of the search interest for the keywords “Vaccine (Argument)”, “COVID-19 Vaccine (Vaccine)” and “COVID Vaccine (Search term)”. Although search trends for these terms were similar, the search volume for the first keyword was considerably greater. As such, we focused further analyses on the keyword “Vaccine (Argument)”. As depicted in Figure 2A, the search interest differed among Italian regions, with the highest value for Tuscany and the lowest for Trentino Alto-Adige. The regional volume of searches for the keyword “Vaccine (Argument)” was moderately but significantly correlated with the regional number of vaccine doses administered per 100,000 inhabitants (Spearman’s ρ = 0.489; *p* < 0.01). 

Figure 2B shows the most common arguments and queries related to the search for “Vaccine (Argument)”. In general, Google users searching for “Vaccine (Argument)” were also interested in updates from or about regional services and drug companies. Indeed, most of the related searches were about booking COVID-19 vaccines, and only a few were about their side effects.

### 3.2. The Relationship between Google Trends Data and Vaccinations

**Figure 3:**
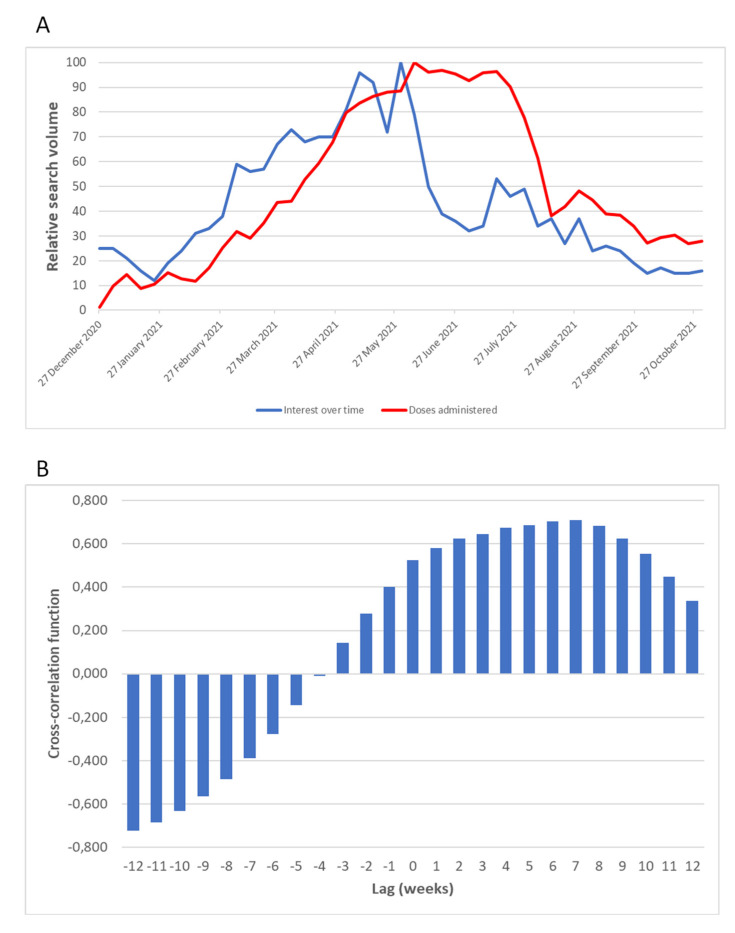
Relationship between Google Trends data and the number of COVID-19 vaccinations. (**A**) Comparison between the overall trends of search interest for the keyword Vaccine (Argument) and the number of vaccine doses administered per 100,000 inhabitants; (**B**) cross-correlation coefficients between the search interest for the keyword Vaccine (Argument) and the number of vaccines administered.

Figure 3A compares the overall trends of data from the Google searches for the keyword “Vaccine (Argument)” with the number of vaccine doses administered per 100,000 inhabitants. The two curves showed a similar shape but were staggered with each other, suggesting a lag between Google Trends data and COVID-19 vaccinations. Indeed, as depicted in Figure 3B, correlation coefficients increased with an increasing number of weeks of delay, reaching a maximum at a 7-week lag.

### 3.3. Prediction of the Trend of Vaccines Administered

**Figure 4:**
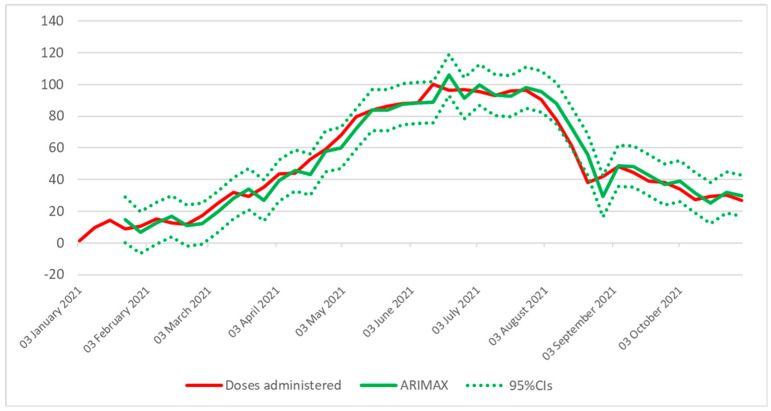
Performance of the ARIMAX model based on Google Trends with a 3-week lag in fitting the number of COVID-19 vaccinations.

Next, several ARIMA models were applied to the overall trend of COVID-19 vaccine doses administered. To meet the assumption of stationarity, the dependent variable was transformed by first-order differencing, and hence the d term in the ARIMA notion was equal to 1. According to the ACF and PACF plots (Supplementary Figure S1), possible values for p and q terms were 0 and 1, respectively. Based on these parameters, three models were fitted, even if ARIMA (1,1,1) produced a non-significant p-value and was discarded. Among those with a significant p-value, ARIMA (0,1,1) performed slightly better than ARIMA (1,1,0) and was therefore chosen for further modelling. Next, Google Trends data were included as an exogenous variable in several ARIMAX models with different lag periods (Supplementary Table S2). All the ARIMAX models with 0–3 weeks of lag had significant p-values for each component. However, the model based on Google Trends with a 3-week lag showed the best performance in fitting the number of COVID-19 vaccinations over time (Figure 4). Compared to the other models, this model indeed had the lowest values for RMSE, MAPE and MAE (Supplementary Table S2).

### 3.4. Changes in Google Search Interest on Vaccination during Pregnancy

**Figure 5:**
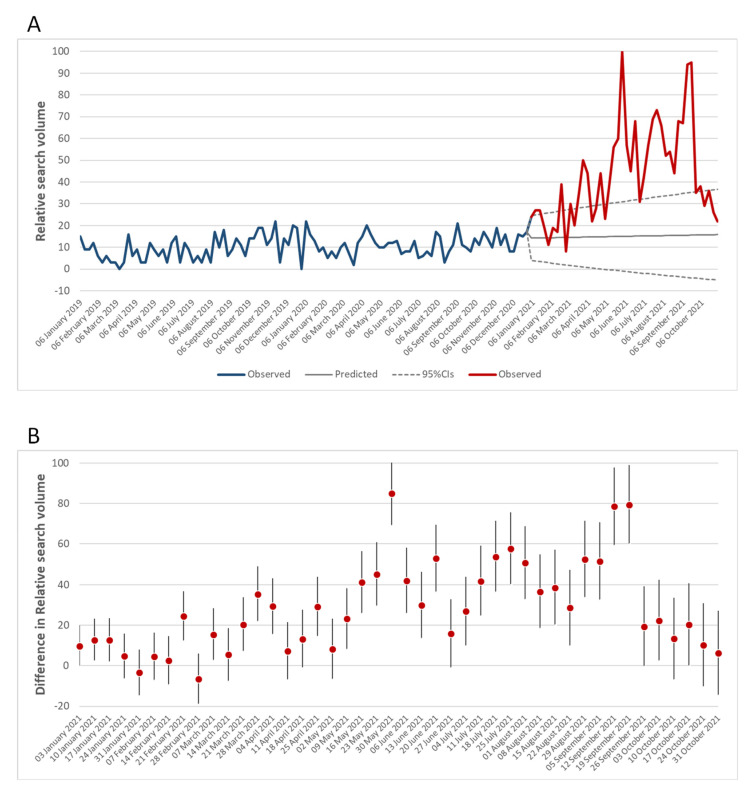
Search interest for the keyword “Vaccine in pregnancy” (Search term) before and after the introduction of COVID-19 vaccination. (**A**) Comparison between observed and predicted values of the search interest for the keyword “Vaccine in pregnancy”; (**B**) differences between weekly observed and predicted values.

**Figure 6:**
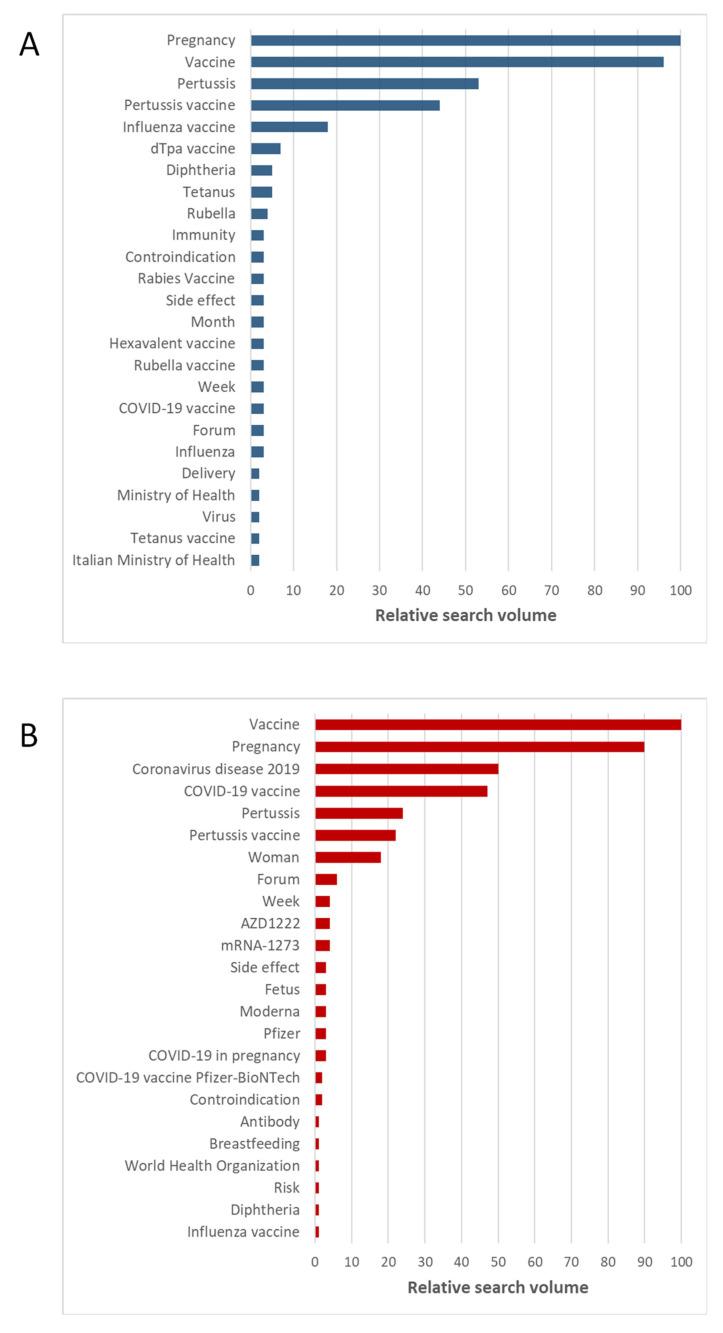
Arguments related to the search activity for the keyword “Vaccine in pregnancy” (Search term). (**A**) Most common arguments before the introduction of COVID-19 vaccination; (**B**) most common arguments after the introduction of COVID-19 vaccination.

Figure 5A shows the overall trend of the search interest for the keyword “Vaccine in pregnancy (Search term)” from 1 January 2019 to 31 October 2021. Notably, the search interest increased after the launch of the COVID-19 vaccination campaign in Italy (i.e., 27 December 2020). In keeping with this, the comparison between observed and predicted values showed significant differences, which reached a peak at the end of May 2021 (Figure 5B). The observed increase in the search interest for “Vaccine in pregnancy (Search term)” followed that shown in Figure 1 for “Vaccine (Argument)” and coincided with the extension of vaccination to all age groups in Italy. The second peak, observed in the middle of September 2021, coincided with the public debate on the COVID-19 green certificate and the administration of booster doses. From a qualitative point of view, we compared the most common arguments related to the search for “Vaccine in pregnancy (Search term)” before and after the introduction of COVID-19 vaccines. Apart from more general arguments (pregnancy, vaccine, etc.), prior to the introduction of COVID-19 vaccines, most of the related searches were about influenza and diphtheria, tetanus and pertussis (dTpa) vaccines (Figure 6A). However, from 1 January 2021, the search interest in COVID-19 and the COVID-19 vaccine increased, becoming two of the most common arguments related to the search for “Vaccine in pregnancy (Search term)” (Figure 6A).

## 4. Discussion

The current COVID-19 pandemic has highlighted the need for innovative approaches and tools for monitoring and forecasting scopes. While initial efforts were directed at predicting the epidemic curve and evaluating the impact of strategies for prevention and control through traditional epidemic models [20,21,22,23,24,25,26,27], later studies emphasised the role of digital and internet surveillance. For instance, the correlation observed between search volumes of COVID-19 terms and epidemiological data has allowed the prediction of a number of infected cases through Google Trends data [12,13,14]. It was also demonstrated that the search volume of vaccine-related terms increased at the beginning of 2020, with some peaks coinciding with public declarations and announcements about new vaccine releases or a significant rise in COVID-19 cases [15]. To our knowledge, however, no study has attempted to investigate the relationship between Google Trends data and vaccine uptake. 

In the present study, we found moderate to strong correlations between the search volume of vaccine-related terms and the number of vaccine doses administered in Italy. Interestingly, there was a lag between the two curves, where the Google Trends data predated the trend of COVID-19 vaccinations by three to seven weeks. This evidence is extremely important because such a lag provides a time window that would allow for promoting vaccination in the event of low vaccine uptake. On the other hand, the observed lag would also allow for better resource allocation in anticipation of growing demand. A similar lag pattern was observed in previous studies [10,13], providing an advantage over conventional surveillance models. Despite this advantage, it was not our intention to use exclusively Google Trends data, but rather to combine them with traditional surveillance systems. Thus, we proposed including Google Trend data in an ARIMAX model for improving the prediction of COVID-19 vaccinations. Among all the models evaluated, the one based on Google Trends with a 3-week lag showed the best performance in fitting the number of COVID-19 vaccinations over time. This finding emphasises the importance of Google Trends analysis and suggests that it can be a useful addition to existing methods of monitoring vaccination uptake and coverage at the national level. 

Our work also aimed to explore if Google Trends might be valid for monitoring changes in feelings associated with the introduction of new vaccines. To do that, we focused on pregnant women because they are more inclined to be worried for themselves and their children [28]. In fact, pregnant women represent a vulnerable group that deserves attention when planning a new vaccination campaign, through proper information on the risks and benefits of vaccination. Yet, contradictory communication from the authorities—often driven by the exclusion of pregnant women from preapproval trials—increased vaccine hesitancy [29]. The main concerns about COVID-19 vaccination relate to doubts about its effectiveness and the fear of side effects [30]. This has resulted in high levels of vaccine hesitancy, especially among more deprived communities and ethnic minority groups [31,32]. Our analysis of Google Trends showed that the introduction of COVID-19 vaccines affected the search interest for the general argument “vaccination in pregnancy” both quantitatively and qualitatively. In particular, there was a significant increase in the search interest after the launch of the COVID-19 vaccination campaign in Italy. Although this evidence was not unexpected, we hypothesised that some changes in the search interest for this argument would coincide with specific events during the vaccination campaign (the extension of vaccination to all age groups, the request for the COVID-19 green certificate, etc.). Moreover, qualitative analysis suggested that this increase was also probably due to concerns about COVID-19 vaccines. These findings, albeit in a general way, might be indicative of anti-vaccination attitudes or vaccine hesitancy in a specific category of people. An in-depth analysis by integrating data from Google Trends and other sources (e.g., specific surveys, Twitter and Facebook Analytics) could provide meaningful insights into the reasons behind vaccine hesitancy among pregnant women. Moreover, a similar approach could also be applied to other at-risk groups (e.g., healthcare workers and patients with chronic diseases) and could be useful for monitoring feelings about vaccination at the population and subpopulation levels. 

It is important to note that our approach has some limitations. Firstly, there are different vaccine-related terms that could show varying strengths of correlation with vaccine uptake. The same is true for internet searches for vaccination in pregnancy. In our study, we chose terms with search volumes that were greater than others while showing a similar trend. Secondly, search terms and Google user behaviours could change as the pandemic scenario evolves, making it necessary to continually update the model. This was particularly true for the qualitative analysis on the search interest for the argument “vaccination in pregnancy”. In fact, the observed increase in search interest was not unexpected and could be simply explained by the interest raised by the introduction of a “new” vaccine. However, a routinary analysis of Google Trends might provide insights into attitudes towards COVID-19 vaccination, vaccine hesitancy and the intention to take the vaccine in specific subgroups. Thirdly, Google Trends does not provide data for all cities; thus, it would be difficult to develop an appropriate model at the local level. Similarly, its data are not stratified by age, gender and other characteristics, making it impossible to analyse findings within specific subgroups. This is also true for pregnant women, although it would have been interesting to compare Google search interest with the number of COVID-19 vaccines administered during pregnancy. 

## 5. Conclusions

Our study suggests the benefits of using Google Trends data to predict the number of COVID-19 vaccine doses administered, and to monitor feelings about vaccination. Interestingly, since the information present in Google Trends precedes the vaccination uptake rate in the general population, these data could prove important for guiding resource allocation and promoting communication strategies when necessary. Moreover, Google Trends provides insights into the fears and concerns among specific groups of people, which should be taken into consideration when planning vaccination campaigns.

## Data Availability

Data that support the findings of this study are available from the corresponding author, upon reasonable request.

## Supplementary Materials

The following supporting information can be downloaded at: https://www.mdpi.com/article/10.3390/vaccines10010119/s1, Table S1. The RECORD statement—checklist of items, extended from the STROBE statement, that should be reported in observational studies using routinely collected health data. Table S2. Summary of the ARIMA and ARIMAX model evaluated. Figure S1. Autocorrelation Function (ACF) and Partial Autocorrelation Function (PACF) plots.

## References

[1] European Centre for Disease Prevention and Control COVID-19 Vaccination. https://www.ecdc.europa.eu/en/covid-19/prevention-and-control/vaccines.

[2] World Health Organization (2021). Monitoring COVID-19 Vaccination: Considerations for the Collection and Use of Vaccination Data.

[3] Cutts F.T., Claquin P., Danovaro-Holliday M.C., Rhoda D.A. (2016). Monitoring vaccination coverage: Defining the role of surveys. Vaccine.

[4] Anema A., Kluberg S., Wilson K., Hogg R.S., Khan K., Hay S.I., Tatem A.J., Brownstein J.S. (2014). Digital surveillance for enhanced detection and response to outbreaks. Lancet Infect. Dis..

[5] Brownstein J.S., Freifeld C.C., Madoff L.C. (2009). Digital disease detection--harnessing the Web for public health surveillance. N. Engl. J. Med..

[6] Eysenbach G. (2011). Infodemiology and infoveillance tracking online health information and cyberbehavior for public health. Am. J. Prev. Med..

[7] Mavragani A., Ochoa G., Tsagarakis K.P. (2018). Assessing the Methods, Tools, and Statistical Approaches in Google Trends Research: Systematic Review. J. Med. Internet Res..

[8] Guo P., Zhang J., Wang L., Yang S., Luo G., Deng C., Wen Y., Zhang Q. (2017). Monitoring seasonal influenza epidemics by using internet search data with an ensemble penalized regression model. Sci. Rep..

[9] Ginsberg J., Mohebbi M.H., Patel R.S., Brammer L., Smolinski M.S., Brilliant L. (2009). Detecting influenza epidemics using search engine query data. Nature.

[10] Shin S.Y., Seo D.W., An J., Kwak H., Kim S.H., Gwack J., Jo M.W. (2016). High correlation of Middle East respiratory syndrome spread with Google search and Twitter trends in Korea. Sci. Rep..

[11] Bragazzi N.L., Alicino C., Trucchi C., Paganino C., Barberis I., Martini M., Sticchi L., Trinka E., Brigo F., Ansaldi F. (2017). Global reaction to the recent outbreaks of Zika virus: Insights from a Big Data analysis. PLoS ONE.

[12] Mavragani A., Gkillas K. (2020). COVID-19 predictability in the United States using Google Trends time series. Sci. Rep..

[13] Kurian S.J., Bhatti A.U.R., Alvi M.A., Ting H.H., Storlie C., Wilson P.M., Shah N.D., Liu H., Bydon M. (2020). Correlations Between COVID-19 Cases and Google Trends Data in the United States: A State-by-State Analysis. Mayo Clin. Proc..

[14] Sulyok M., Ferenci T., Walker M. (2021). Google Trends Data and COVID-19 in Europe: Correlations and model enhancement are European wide. Transbound. Emerg. Dis..

[15] Pullan S., Dey M. (2021). Vaccine hesitancy and anti-vaccination in the time of COVID-19: A Google Trends analysis. Vaccine.

[16] Klugar M., Riad A., Mekhemar M., Conrad J., Buchbender M., Howaldt H.P., Attia S. (2021). Side Effects of mRNA-Based and Viral Vector-Based COVID-19 Vaccines among German Healthcare Workers. Biology.

[17] Riad A., Schünemann H., Attia S., Peričić T.P., Žuljević M.F., Jürisson M., Kalda R., Lang K., Morankar S., Yesuf E.A. (2021). COVID-19 Vaccines Safety Tracking (CoVaST): Protocol of a Multi-Center Prospective Cohort Study for Active Surveillance of COVID-19 Vaccines’ Side Effects. Int. J. Environ. Res. Public Health.

[18] Benchimol E.I., Smeeth L., Guttmann A., Harron K., Moher D., Petersen I., Sørensen H.T., von Elm E., Langan S.M., Committee R.W. (2015). The REporting of studies Conducted using Observational Routinely-collected health Data (RECORD) statement. PLoS Med..

[19] Box G. Time Series Analysis: Forecasting and Control; Palgrave Macmillan, London, UK, 2015.

[20] Wu J.T., Leung K., Leung G.M. (2020). Nowcasting and forecasting the potential domestic and international spread of the 2019-nCoV outbreak originating in Wuhan, China: A modelling study. Lancet.

[21] Boldog P., Tekeli T., Vizi Z., Dénes A., Bartha F.A., Röst G. (2020). Risk Assessment of Novel Coronavirus COVID-19 Outbreaks Outside China. J. Clin. Med..

[22] Giordano G., Blanchini F., Bruno R., Colaneri P., Di Filippo A., Di Matteo A., Colaneri M. (2020). Modelling the COVID-19 epidemic and implementation of population-wide interventions in Italy. Nat. Med..

[23] Gatto M., Bertuzzo E., Mari L., Miccoli S., Carraro L., Casagrandi R., Rinaldo A. (2020). Spread and dynamics of the COVID-19 epidemic in Italy: Effects of emergency containment measures. Proc. Natl. Acad. Sci. USA.

[24] Maugeri A., Barchitta M., Battiato S., Agodi A. (2020). Estimation of Unreported Novel Coronavirus (SARS-CoV-2) Infections from Reported Deaths: A Susceptible-Exposed-Infectious-Recovered-Dead Model. J. Clin. Med..

[25] Kucharski A.J., Klepac P., Conlan A.J., Kissler S.M., Tang M.L., Fry H., Gog J.R., Edmunds W.J., Emery J.C., Medley G. (2020). Effectiveness of isolation, testing, contact tracing, and physical distancing on reducing transmission of SARS-CoV-2 in different settings: A mathematical modelling study. Lancet Infect. Dis..

[26] Maugeri A., Barchitta M., Battiato S., Agodi A. (2020). Estimation of unreported SARS-CoV-2 cases in Italy using a Susceptible-Exposed-Infectious-Recovered-Dead model. J. Glob. Health.

[27] Maugeri A., Barchitta M., Battiato S., Agodi A. (2020). Modeling the Novel Coronavirus (SARS-CoV-2) Outbreak in Sicily, Italy. Int. J. Environ. Res. Public Health.

[28] Barchitta M., Maugeri A., Magnano San Lio R., La Rosa M.C., La Mastra C., Favara G., Giunta G., Cianci A., Agodi A. (2021). Vaccination Status of Mothers and Children from the ‘Mamma & Bambino’ Cohort. Vaccines.

[29] Abbas-Hanif A., Modi N., Smith S.K., Majeed A. (2021). Covid-19 treatments and vaccines must be evaluated in pregnancy. BMJ.

[30] Kilich E., Dada S., Francis M.R., Tazare J., Chico R.M., Paterson P., Larson H.J. (2020). Factors that influence vaccination decision-making among pregnant women: A systematic review and meta-analysis. PLoS ONE.

[31] Kamal A., Hodson A., Pearce J.M. (2021). A Rapid Systematic Review of Factors Influencing COVID-19 Vaccination Uptake in Minority Ethnic Groups in the UK. Vaccines.

[32] Cascini F., Pantovic A., Al-Ajlouni Y., Failla G., Ricciardi W. (2021). Attitudes, acceptance and hesitancy among the general population worldwide to receive the COVID-19 vaccines and their contributing factors: A systematic review. EClinicalMedicine.

